# Review of adult gender transition medications: mechanisms, efficacy measures, and pharmacogenomic considerations

**DOI:** 10.3389/fendo.2023.1184024

**Published:** 2023-07-04

**Authors:** Inder Sehgal

**Affiliations:** Department of Biomedical Sciences, Rocky Vista University, Ivins, UT, United States

**Keywords:** transgender, gender dysphoria, pharmacogenetics, hormonal therapy, estradiol, testosterone, spironolactone, pharmacodynamics

## Abstract

Gender dysphoria is the imparity between a person’s experienced gender and their birth-assigned gender. Gender transition is the process of adapting a person’s sexual characteristics to match their experienced gender. The number of adults receiving sex hormone therapy for gender dysphoria is increasingly and these pharmacotherapies are increasing being prescribed in a general practice setting. The role of hormone therapy is to reverse or reduce physical sexual characteristics of the birth-assigned gender and enhance and build characteristics aligning to the expressed gender and these therapies apply to both transgender and gender nonconforming patients. Recognizing the options and interpreting the effects of gender transition therapies are fundamental to the discussion and treatment of gender dysphoria. This review summarizes pharmacodynamics, comparative dosing, adverse effects, monitoring, and potential pharmacogenetic influence of current pharmacotherapy. These include the use of 17-beta-estradiol, spironolactone, testosterone, GnRH agonists as well as adjunctive phosphodiesterase-5 inhibitors. The article also addresses gaps within the published literature including optimal routes of administration for individual patients, risks of malignancy and dosing reductions as transgender patients age.

## Introduction

Gender dysphoria is the imparity between a person’s experienced or expressed gender and their birth-assigned gender, which is based on primary or secondary sexual characteristics (1). Gender transition is the process of adapting a person’s sexual characteristics to match the sex with which they identify. Adults who undergo gender transition or who are gender nonconforming may utilize hormone therapy to reverse or reduce physical sexual characteristics of their birth-assigned gender as well as to develop and affirm characteristics of the transgender sex (1). The number of adults receiving sex hormone therapy for gender dysphoria is increasing at both specialty and general practice settings (2). Although over the past two decades gender transitioning individuals have been approximately two to one Male to Female (MTF) rather than Female to Male (FTM), recent data point to equivalent numbers initiating sex hormone therapy (3). Several protocols for gender transition therapies exist; however, the quality of evidence supporting the therapies is low and optimal target ranges have not been established for serum sex steroids levels (4, 5). Current best practices for gender transitioning pharmacotherapy is commonly referenced from two sources, the World Professional Association for Transgender Health (5) Standards of Care, and the recommendations published by the Endocrine Society. This review will summarize best practices from these and other sources from the perspective of comparative dosing with other hormone therapies. It will identify the pharmacodynamics, adverse reactions, and monitoring for recommended medications used for gender transitions both MTF and FTM. In addition, this review will include adjunctive therapies such as phosphodiesterase-5 inhibitors. The role of pharmacogenomics has not been included in routine pharmacotherapies for gender dysphoria and this review introduces mechanisms through which pharmacogenomics could influence and explain individual patient responses to estrogen use.

Hormonal therapy in gender transition has two pharmacodynamic goals. The first is to reduce endogenous sex hormone levels leading to the reduction of expressed secondary sex characteristics. The second goal is to add exogenous sex hormones consistent with the gender to be affirmed so that plasma levels for exogenous hormones are within the normal physiologic range for the affirmed gender (4). Pharmacologic hormonal therapies are followed through monitoring of plasma levels, secondary sex characteristic changes and potential adverse reactions. The adverse reactions these hormonal therapies may present occur both short and long term.

## Feminizing hormone regimes

The strategy for transgender women is to suppress androgen signaling to reduce male secondary sex characteristics and add estrogen to enhance the female phenotype. Androgen suppression is accomplished through both the negative hypothalamic-pituitary feedback arising from estrogen supplements as well as direct androgen signaling block using receptor antagonists. Pituitary suppression of Follicle stimulating hormone (FSH) and Luteinizing hormone (LH) using a GnRH agonist is also an option. 17-beta-estradiol is the estrogen now recommended to feminize the patient’s external appearance (5). Mechanistically, it binds to estrogen receptors in estrogen responsive organs, where it regulates gene expression, leading to feminized appearance. In addition, estradiol suppresses pituitary LH leading to reduced gonadal testosterone synthesis. Estrogen therapy alone can, through negatively feedback, reduce testosterone production in male to female patients down to low-normal ranges for biological males (200–300ng/dL), but not to the normal testosterone levels of natal females (75ng/dL) (6, 7). In most cases, exogenous hormonal therapies will be maintained throughout life (3).

Three routes of administration for 17-beta-estradiol are available: oral, transdermal and intramuscular injections. Oral therapy may be the most common (8) while transdermal estrogen is favored for individuals at greater risk for developing venous thromboembolism (VTE). Although existing data do not call for specific dosing reductions as transgender females age; some epidemiology studies point to the increased incidence of cardiovascular disease and venous thromboembolism (VTE) in transgender females receiving estrogen; therefore, use of transdermal estrogen and anticoagulation agents and a reduction in dosing may be warranted (9). Conjugated estrogens (i.e., ethinyl estradiol) were previously recommended as an estrogenic option but are now not recommended due to increased risk of thromboembolism (TE) with these synthetic estrogens (3).

The dosing of estradiol in all forms given to transgender women and for feminizing therapy is far above typical levels administered to cisgender women (**Table 1**; cisgender refers to a gender identity aligned with the sex assigned at birth). For oral estradiol 17β, transgender women receive 2 – 6 mg, and up to 10 mg/day. Levels used for ciswomen for post-menopausal supplementation, hypogonadism, oophorectomy, and primary ovarian failure dosing are under 2 mg/day. Estradiol used in men for advanced androgen dependent prostate cancer is also under 2 mg/day (5). The dosing for the estradiol transdermal patch is 25 – 200 mcg/day; this 200-mcg upper level is well above the upper level (50 mcg/day) for the patch in postmenopausal women, for hypogonadism, oophorectomy and primary ovarian failures. The injectable estrogens (cypionate and valerate) are administered to transgender women at 5 – 30 mg every 2 weeks while they are dosed for cisgender women every 4 weeks and the maximal dose is under 10 mg (cypionate) and up to 20 mg (valerate) (5).

**Table 1:** Gender Transition Medications: Gender transition dosing in comparison with dosing for other common indications.

Medication	Condition	Typical Dosing	Reference
Spironolactone	Transgender Female	100-200 mg/day (up to 400 mg)	10
	Heart failure with reduced ejection fraction	12.5 – 50 mg PO qd	11
	Hypertension	25 – 100 mg PO qd	
	Primary Hyperaldosteronism	12.5-400 mg PO qd	
Estradiol 17β oral	Transgender Female	2-6 mg/day	5, 10
	Postmenopause	0.5 – 2 mg PO qd	12
	Hypogonadism, Oophorectomy, Primary Ovarian Failure	1-2 mg PO qd	
	Advanced Androgen-Dependent Prostate Cancer	1-2 mg PO tid	
Estradiol Transdermal Extended Release Patch	Transgender Female	0.025-0.2 mg/day	5
	Post menopause	0.0375-0.05 mg q 24 h, applied twice weekly; 0.025 mg/24 h q week	13
	Hypogonadism, Oophorectomy, Primary Ovarian Failure	0.0375-0.05 q 24h twice weekly; 0.025-0.1mg once weekly	
Estradiol Cypionate	Transgender Female	5-30 estradiol cypionate mg IM q 2 weeks; 2-10 mg IM q week	4
	Post menopause (PM); Hypogonadism, Oophorectomy, Primary Ovarian Failure	1-5 mg IM q 3-4 weeks; 1.5-2 mg IM q 4 weeks.	14
Estradiol Valerate	Transgender Female	5-30 mg IM q 2 weeks; 2-10 mg IM q week	4
	Post menopause; Hypogonadism, Oophorectomy, Primary Ovarian Failure	10-20 mg IM q 4 weeks	15
GnRH agonist	Transgender Female	3.75-7.5 mg SQ/IM monthly; 11.25/22.5 mg SQ/IM 3/6 monthly	5
	Prostate Cancer Therapy (Goserelin/Leuprolide)	3.6 mg SQ 28d/7.5 mg IM/SQ q month	16, 17
	Endometriosis Breast Cancer-Palliative (Goserelin/Leuprolide)	3.6 mg SQ q 28d for 6 months/3.75 mg IM monthly for up to 6 months	
Testosterone Enanthate/Cypionate	Transgender Male	50-100 mg IM/SQ weekly/100-200 mg IM q 2weeks/750 mg IM q 10 weeks	5
Testosterone Enanthate/Cypionate	Hypogonadism	50-100 mg IM q week or 100 – 200 mg q 2 weeks	18
Testosterone Transdermal Patch	Transgender Male	2.5-7.5 mg/day	4
	Hypogonadism	2-6 mg/day	18
Testosterone Transdermal Gel 1.62%		50-100 mg/day	4
	Hypogonadism	20.25-81 mg qd	18

PO, orally; SQ, subcutaneously; IM, intramuscularly; q, every; qd, each day; tid, three times each day; x 5 days, for 5 days; h, hours.

Treatment with physiologic doses of estrogen alone is insufficient to suppress testosterone levels into the normal range for cisgender females (4). Therefore, individuals undergoing feminizing hormone therapy require the addition of an anti-androgen medication to further inhibit testosterone signaling either by inhibiting production or by blocking the androgen receptor. In the USA, all forms of estradiol (parenteral, oral, and patches or gels) are combined with androgen suppressive therapy, usually spironolactone (5, 7). Mechanistically, spironolactone is better known as an aldosterone antagonist; however, it also binds to the androgen receptor and in doses of up to 400 mg/day, functions as an androgen receptor antagonist and potentially as an estrogen receptor agonist (7, 19). The androgen receptor blockage prevents virilization and promotes feminization (1). Spironolactone has well-known mechanisms as a potassium-sparing diuretic and therefore patients taking this medication risk hyperkalemia; however, hyperkalemia appears to be an uncommon and transient phenomenon in most transgender women (20). Other potential adverse effects of spironolactone are dehydration and hyponatremia (21).

The dosing of spironolactone for feminizing hormone therapy is 100-300 mg/day (**Table 1**). This is in the range of spironolactone used to treat primary hyperaldosteronism and above the range of dosing for heart failure and hypertension (see references in **Table 1**). As an alternative to spironolactone, gonadotropin-releasing Hormone (GnRH) agonists, leuprolide, histrelin, or goserelin can be used along with the estradiol. GnRH agonists act by stimulating the pituitary GnRH receptors leading to receptor desensitization (5). This desensitization then reduces the secretion of gonadotropins luteinizing hormone (LH) and follicle-stimulating hormone (FSH), which leads to decreased stimulation of testicular testosterone production (7). The upper range of goserelin dosing is approximately double that for cisgender women receiving treatment for endometriosis and for breast cancer as well as double the dose for prostate cancer in men. For leuprolide, transgender women receive the equivalent dose of men treated for prostate cancer (**Table 1**).

No head-to-head studies have been published that establish the superiority of spironolactone over GnRH agonists to lower testosterone. The use of GnRH agonists is limited in many countries because of their cost. For example, in the U.S., a four-week supply of goserelin implant has a cash discount price of $900 vs. approximately $20 for 4 weeks of spironolactone (22). In several European countries, the common regimen includes oral estradiol combined with cyproterone (50 mg daily); however, cyproterone is not used in the U.S. due to its risk of TE (7).

The estradiol and testosterone levels in Male to Female transgender patients should be monitored every 3 months initially, then every 6 – 12 months with the targeted maintenance equivalency of premenopausal females (100 - 200 pg/ml estradiol and 50 ng/dL testosterone (4). The estradiol concentrations should not exceed 400 pg/mL. Spironolactone is monitored via potential adverse effects of hyperkalemia; serum potassium concentrations should be assayed every 3–4 months in the first 2 years and annually after two years (4, 7). Individuals with impaired renal function have the greatest risk of hyperkalemia.

Overall, the greatest risk for an adverse event from feminizing hormone therapy is the development of TE events, such as deep vein thrombosis and pulmonary embolism. Transgender females with any history of prior TE should be evaluated and treated preventatively before beginning estrogen therapy (5). Estrogens affect several hepatic coagulation proteins including elevated factors VII, VIII and fibrinogen and activated protein C (23). The ethinyl chemical group slows estrogen catabolism and inactivation resulting in significantly greater prothrombotic changes to liver proteins. Thus, ethinyl estradiol is no longer recommended for feminizing hormone therapy (4, 7). With the natural estradiol-17β, the lifetime risk of thromboembolism is still about 1% and is more frequent with the use of oral estrogens. Therefore, use of transdermal and injectable estradiol preparations may confer less risk of TE than oral estrogens. Oral dosages first pass through the liver, potentially altering the synthesis of clotting factors, while non-oral routes circumvent the first-pass, and may lead to a lower risk of TE in older transgender females (4).

The block of androgen signaling via estrogen and an antiandrogen such as spironolactone leads to a loss in testicular volume, decreased desire for sex, fewer or absent spontaneous erections and delayed ejaculation (4, 5, 24). Treatment will also reduce sperm quantity and quality, and eventually lead to irreversible infertility; this may occur even if hormonal therapy is halted. These effects are consistent with secondary hypogonadism. Because of the erectile dysfunction, one potential addition to the medication regime is a phosphodiesterse-5 inhibitor (PDE5i), often sildenafil or tadalafil if desired by the patient. The inhibition of PDE5 enhances erection by inhibiting the breakdown of cyclic GMP; cGMP causes smooth muscle relaxation and thus increased cavernosal blood flow (25). Interestingly, these medications are not listed or discussed in the major therapeutic guideline references (4, 5, 7). A recent search on PubMed using terms Transgender and Sildenafil yielded no results; thus, the addition of medications for the erectile dysfunction in transgender females appears to not be well documented in peer-reviewed literature. An overview of feminizing hormone therapy states that both “sildenafil (Viagra) and tadalafil (Cialis) can be used for preservation of erectile function at any stage or with any feminizing hormone regimen” (9); however, the lack of peer-review studies indicates that future research is needed to define the extent and best practices for the use of PD5 inhibitors in the field of transgender pharmacology.

The risk of breast cancer in transgender females has not been extensively followed, however, current data indicate a relative risk above cisgender men but less than cisgender women. Mammography for transgender women with no known increased risk of breast cancer should be at the same age recommended for cisgender women (7). In addition to breast cancer, transgender females also run the risk of prostate cancer and should be monitored through age-appropriate individualized screening according to personal risk (4). Additional parameters for monitoring in patients receiving feminizing hormone therapy with estrogens, are prolactin, fasting lipids and bone density screening. Bone density should be assessed before starting estrogens and at follow-up visits for patients at risk for osteoporosis. For patients without risk factors for osteoporosis, bone density screening should begin be at the same age recommended for cisgender women.

Treatment of postmenopausal women with estrogen, with or without progesterone, has been associated with an increase in triglycerides and HDL cholesterol and a decrease in total and LDL cholesterol (7). Other more moderate risks are potential macroprolactinoma reported in up to 20% of transgender females treated with estrogens.

Hormone therapy for feminization will produce desired physical effects over time. The feminizing physical effects of estradiol plus anti-androgen pharmacotherapies for transition in transgender women will start within 3 to 12 months and progress over the next three years (7). These therapies will lead to decreased facial and body hair, increased subcutaneous fat, decreased lean body mass, redistribution of body fat deposits and decreased skin oil. Breast tissues will grow to an extent; however, fewer than 20% of transgender females will achieve Tanner breast stages 4–5 (26). Most will develop up to Tanner Stage 2 and any development will be achieved in 2 years. For this reason, approximately two-thirds of transgender females opt for surgical breast augmentation (27). No pharmacologic treatments have definitively proven to enhance breast development in transgender women; however, high-dose cimetidine (>1000mg/day) over a chronic period is reported to cause breast hypertrophy (6).

Hormone therapies for transgender women will be used long-term and they offer several health benefits in addition to the risk of VTE. Estrogen preserves bone density although osteoporosis screening should begin as recommended for cisgender women (currently age 60). One prospective study of transgender females also found favorable changes in patient’s plasma lipid profiles, as high-density lipoprotein increased and low-density lipoprotein decreased (4). Spironolactone therapy over a chronic period at elevated dosing similar to that used for gender transitioning offers the benefit of reduced risk of prostate malignancy; (28) thus, spironolactone could reduce this risk in transgender females as well although no clinical studies have yet documented this risk reduction. Current practice guidelines are for transgender female patients to be screened for breast and prostate cancer appropriately (10).

## Masculinizing hormone regimes

Masculinizing hormone therapy relies largely on testosterone administration (4). Testosterone is administered either by intramuscular or subcutaneous injection or transdermally with either patch or gel and with a plasma target of 320 to 1000 ng/dL. The dosing levels for testosterone used for transitioning in transgender males fall within the lower end of the range used for hypogonadism in cisgender males and while subcutaneous administration is used for gender-affirming hormone therapy, testosterone is used IM for hypogonadism. The masculinizing dose of the undecanoate is 750 mg intramuscularly (IM) every 10 weeks vs. 750 mg IM three times within 10 weeks for hypogonadism (**Table 1** and references); for enanthate or cypionate, 50 – 200 mg weekly for TG men vs. up to 400 mg for hypogonadism. For the undecanoate, due to the risk of oil microembolism, this form is administered in a clinical facility (29). As opposed to the lower dosing compared with hypogonadism for injectable testosterone, the testosterone patch dosing and gel dosing ranges are above that for hypogonadism (**Table 1**).

Testosterone is metabolically converted into dihydrotestosterone, which is the most biologically active ligand for the intracellular androgen receptor. Activation of the receptor induces transcription or repression of androgen-regulated gene expression. Physical changes resulting from testosterone activity occur in stages. Between one to 6 months, menses ceases, and desired effects of facial, chest, abdominal hair increase. Skin oil increases along with muscle mass and there is redistribution of fat. Transgender males also experience increased sexual desire. Over year one, the individual’s voice deepens, they develop clitoromegaly, and can experience male pattern baldness loss if genetically predisposed (4, 6). Testosterone leads to a decrease in breast glandular tissue and overall adipose tissue, as well as an increase in breast fibrous connective tissue. The resultant mammary tissue histologically resembles postmenopausal breast tissue (6). If breakthrough uterine bleeding occurs, depot medroxyprogesterone (150 mg every 3 months) may be an option (30).

Erythrocytosis as a result of testosterone therapy in transgender males appears to be the most significant health risk. This rise in hematocrit (31) are due to testosterone and dihydrotestosterone (DHT) stimulation of the production of erythropoietin leading to increased erythropoiesis (32). The increased hematocrit increases blood viscosity, as well as platelet adhesiveness raising the potential for TE due to hyperviscosity (33). Testosterone therapy also increases the risk for adverse cardiovascular events, including myocardial infarction, hypertension, decreased HDL-cholesterol, elevated triglycerides, and low-density lipoprotein cholesterol and excess weight (5). The elevated cardiovascular risk comes through increased coronary artery and cerebrovascular disease due to the more atherogenic lipid profile (4). Hypertension can occur because of increased salt retention, and this typically occurs within 2 to 4 months following the initiation of testosterone therapy (34). Transgender therapy with testosterone will lead to a temporary or permanent decreased or lost fertility. Transgender men also have a moderate risk of severe liver dysfunction (as measured by transaminases), breast or uterine cancer (4). Another adverse sign is androgenic acne (5).

## Pharmacogenomic considerations

Pharmacogenetic interactions have not been investigated at the dosing levels of hormone medications used for gender transition. Available data indicate that the genetic polymorphisms that influence spironolactone efficacy in reducing blood pressure (35), act via enhancing or decreasing pathways specific to hypertension (36, 37) and would not influence response in transgender pharmacodynamics. Testosterone has no clinical annotations which report an association between a variant and a drug phenotype from a publication (35).

Estrogen, however has allelic variances associated with receptor polymorphisms and conceivably, these same alterations could modify responses to estrogens when used to assist gender transition. There are two main types of estrogen receptors (ERs): estrogen receptor alpha (ERα) or estrogen receptor 1 (ESR1), and estrogen receptor beta (ER-β) or estrogen receptor 2 (ESR2). Both of these are nuclear receptors activated by the sex hormone, estrogen. In natal females, ESR1 is mostly active in the mammary gland and uterus, and aides in the regulation of skeletal homeostasis and metabolism. In natal males, ESR1 is found on reproductive tissues such as the testes, vas deferens, seminal vesicles, efferent ductules, and prostate, while ESR2 is found in the interstitial cells, germ cells, Sertoli cells and efferent ductules. ESR1 is also expressed in natal males in skeletal muscle, beta pancreatic cells, bone, cardiac ventricles, vascular smooth muscle, brain, lymphocytes, and macrophages. This distribution leads to a physiologic role of estrogens in men for bone growth, glucose and lipid metabolism, and FSH and LH concentrations (38).

Several variants for the ESR1 gene have been reported (39) and clinical annotations exist for the estrogen receptor type 1. Patients with variant rs9340799, a SNP yields either A or G nucleotides in the estrogen receptor 1 gene. Cisgender females with the AA genotype or heterozygote AG genotypes may experience smaller responses to estrogens as measured by maintenance of spine bone mineral density as compared to GG phenotype patients. Patients with the GG genotype may experience greater increases in spine bone mineral density. The A allele is distributed is most populations and in over three-quarters of East Asians, Finnish people, African/African Americans and Latinos (35). No study has yet investigated if the presence of AA or the GG alleles in transgender females is associated with desired or adverse responses to estrogens. However, the divergent physiologic effects of ESR1 variants on bone mineral density in cisgender females indicate estrogen receptor genomics can shape the pharmacologic response to estrogen. Future studies into the pharmacogenetics of gender transition estrogens are needed to address questions such as if ESR1 variants expressed in sex organs of males may shape the degree of sexual disfunction and bone density in transgender females. A recent study has reported that another polymorphism of the ESR1 gene encoding shorter than average numbers of repeats are overrepresented in transgender men (40). The study did not assess therapeutic response to estrogen therapies, but indicate that ESR1 variants associate with the dysphoric condition.

## Conclusion

Transgender patients and gender nonconforming patients are prescribed hormone agonists for replacement pharmacotherapy and often antagonists that result in short and long-term desired and adverse effects. For feminizing therapy, the levels of estradiol administered are often more than four times levels used in cisgender women for hormone therapies and the dosing of spironolactone is equivalent to levels used to treat edema. Testosterone therapy for transgender men is equal to or above testosterone dosing for treatment of male hypogonadism. Both estradiol and testosterone are administered to reach plasma drug concentrations equivalent to the physiologic range for the experienced or expressed gender. The long-term effects, both physiologic and potentially pathologic, of these elevated hormone levels warrant further investigation (6).

Although the pharmacodynamic mechanisms differ, both estradiol and testosterone raise the risk of thromboembolic events in transgender patients. Both hormones however also preserve bone density (41). Long-term monitoring for transgender individuals long term currently may include prostate and breast cancer screening, lipid profiling, liver function assays, hematocrit (for erythrocytosis), hypertension and coronary artery disease screening (4).

There are notable gaps within the published literature on transgender pharmacology that could be addressed through surveys or case-control or cohort trials. These include identifying which routes of administration are most frequent in primary care practice and what criteria is used to determine the optimal route for individual patients. Other gaps raise the questions of: Is estrogen dosing reduced in aging transgender females and if so, to what reduced levels? Is there a shift away from oral estradiol in patients with increased age or development of TE risk or addition of other anticoagulation medications? What is the frequency of prescribing a PDE5 inhibitor for erectile dysfunction in transgender females? Further investigation can also help define the presence of pharmacogenetic responses to estradiol due to ESR1 genomic variants.

A recent Cochrane database search in 2019 investigated randomized controlled trials (RCTs), quasi-RCTs, or cohort studies that enrolled transgender women, age 16 years and over who were undergoing transition pharmacotherapies (1). The search investigated antiandrogen and estradiol used alone or in combination. Seven hundred, eighty-seven references were initially identified but all were excluded because they could not meet inclusion criteria. The most common reason for the exclusion was the lack of an adequate control group. The authors concluded therefore that insufficient evidence currently exists in the literature to determine the efficacy or safety of hormonal treatment for transgender women. This gap in evidence also points to the need for trials to examine types of antiandrogens, dosing and routes of administration with estradiol alone. Outcomes should include quality of life, monitoring and adverse responses. In addition, although there is concern surrounding the use of prolonged hormone therapies with increasing the relative risk of certain malignancies, current published literature does not sufficiently address this risk (6).

With regard to breast cancers, the Endocrine Society recommends against the use of testosterone replacement therapy in cisgender males who develop breast cancer (42). In some cisgender females with breast cancers, testosterone is one option for therapy, although because testosterone is aromatized into estrogen, the risk exists for elevation of estrogenic breast cancer growth as well (43). The continued use of testosterone in transgender males who develop breast cancer is an open question and concern. A recent systematic literature review of published data in transgender males found a greater incidence of breast cancer in transgender men on testosterone therapy than in cisgender men also on testosterone replacement; however, the data numbers were too low to allow for recommendations to halt testosterone therapy in these men (44).

The field of transgender/gender nonconforming pharmacy including pharmacology, monitoring, adverse effects, geriatric modifications and pharmacogenetics is expanding in scope and depth. With greater numbers of patients come great opportunities for more robust cohort and case-controlled studies, and future research will without doubt lead to evolution of best practices. These best practices should be instructed in the curricula of practitioners such as physicians, pharmacists nurse practitioners and physician assistants. This process may be gradually occurring although presently, the instruction of transgender pharmacology and pharmacotherapy is not widely incorporated into professional education texts. Of four major pharmacotherapeutic sources, Lehne’s Pharmacotherapeutics for AP Nurses and P.A.s (45), Pharmacotherapy, A Pathophysiologic Approach (46), Pharmacotherapy Principles and Practice (47), only Pharmacotherapeutics for Advanced Practice Nurse Prescriber (48) includes a separate chapter on transgender patients.

Many aspects of pharmacotherapy for transitioning can be accomplished by primary care providers; however, because of needs for monitoring, knowledge of adjunct medication options, and training environments, it has been suggested that gender-affirming hormone therapy may be best managed in consultation with experts who specialize in the care and treatment of transgender individuals (49). However, all who are involved in transgender pharmacy and therapy benefit from knowledge of current best practices, monitoring, expected responses, potential adverse responses and future directions needed to fill in knowledge gaps in therapy.

## Author contributions

The author confirms being the sole contributor of this work and has approved it for publication.
