# Corrigendum: Review of adult gender transition medications: mechanisms, efficacy measures, and pharmacogenomic considerations

**DOI:** 10.3389/fendo.2024.1537014

**Published:** 2025-01-06

**Authors:** Inder Sehgal

**Affiliations:** Department of Biomedical Sciences, Rocky Vista University, Ivins, UT, United States

**Keywords:** transgender, gender dysphoria, pharmacogenetics, hormonal therapy, estradiol, testosterone, spironolactone, pharmacodynamics

In the published article, there was an error. The claim made by a referenced citation may not be supported by the literature.

A correction has been made to **Feminizing hormone regimes,** Paragraph 9.

This sentence previously stated: “Treatment will also reduce sperm quantity and quality, and eventually lead to irreversible infertility; this will occur even if hormonal therapy is halted (7)”.

The corrected sentence appears below: “Treatment will also reduce sperm quantity and quality, and eventually lead to irreversible infertility; this may occur even if hormonal therapy is halted”.

The authors apologize for this error and state that this does not change the scientific conclusions of the article in any way. The original article has been updated.

